# The Role of the *Matrix Metalloproteinase-9* Gene in Tumor Development and Metastasis: A Narrative Review

**DOI:** 10.1055/s-0043-1768166

**Published:** 2023-04-17

**Authors:** Datis Kalali

**Affiliations:** 1 Medical School, University of Cyprus, Nicosia, Cyprus

**Keywords:** matrix metalloproteinases, MMP-9, carcinogenesis, metastasis, oncogenes

## Abstract

Matrix metalloproteinase-9 (MMP-9) is one of the widely studied enzymes of the extracellular matrix which can degrade various matrix biomolecules. The gene coding for this enzyme has been found to be associated with various multifactorial diseases, including cancer. More specifically, the expression of MMP-9 and polymorphisms of its gene have been found to be correlated with the formation and the invasiveness of different types of cancer. Hence, the latter gene can potentially be used both as a clinical genetic marker and a possible target in anticancer therapy. The present minireview explores the role of the *MMP-9* gene in the process of tumor formation, growth, and metastasis and presents an overview of the polymorphisms of the gene associated with cancer as well as its regulation mechanisms, to provide an insight into the potential clinical applications. Nevertheless, further clinical trials and research are still required to reach more valuable conclusions for the clinical implications of the recent findings.

## Introduction

Matrix metalloproteinases are a group of enzymes that can degrade various proteins and protein derivatives found in the extracellular matrix (ECM). 1 One of the widely studied enzymes of this family is matrix metalloproteinase-9 (MMP-9), which is capable of degrading type IV collagen, a basic structure of the ECM, as well as other substrates such as angiotensin II and plasminogen which are not ECM proteins. 2 3 The *MMP-9* gene which codes for the MMP-9 enzyme is found in the genetic location 20q13.12 and has been widely studied in different hereditary and multifactorial diseases, including renal and ophthalmic disorders. 4 5 6

With cancer remaining a leading cause of mortality despite the novel discoveries in the field of oncology, it is necessary to study the molecular biology of cancer in more depth with the hope of discovering information which can assist the diagnosis and treatment of the disease. 7 One of the major factors that impact the development, progression, and therapeutic procedure of solid carcinomas is the tumor microenvironment, which includes the ECM and its proteins. 8 9 On the contrary, several research has indicated that the ECM and the enzymes which assist its formation and degradation, such as matrix metalloproteinases, play a significant role in the process of oncogenesis and indeed genetic mutations in them can be a risk factor for higher predisposition to many types of cancer. 10 11 12 13 With this rationale, the gene coding for MMP-9 can potentially be studied as a genetic marker of cancer as well as a possible target for cancer prevention and early-stage therapy. Moreover, it is known that for a solid tumor to migrate, it must first invade the ECM, and therefore, its enzymes are a major factor that can influence the metastatic progression of carcinomas. 14 More specifically, several studies have validated that the expression of MMP-9 is associated with a higher risk of metastasis and a lower survival expectancy in different types of cancer. 15 16 17

Hence, MMP-9 is indeed a significant gene in different malignant neoplastic diseases and its multifactorial role can provide more insights into diagnostics and therapeutics in the field of medical oncology. Given that the evidence on the role of the *MMP-9* gene in cancer is scattered in different research articles, the present narrative review aims to provide an overview of the available evidence, including its role in carcinogenesis and tumor migration, the polymorphisms of the gene associated with cancer, as well as its clinical significance.

## The Role of Matrix Metalloproteinase-9 in Carcinogenesis and Metastasis

### Matrix Metalloproteinase-9 as Carcinogen and Prosurvival Protein in Cancer

Due to its wide range of substrates, MMP-9 can interfere with various biological mechanisms of the organism which can subsequently trigger the process of tumorigenesis directly or indirectly. 18 To begin with, MMP-9 can activate different proteins involved in the inflammatory pathways and thus act as a proinflammation factor. 19 20 Chronic inflammation is known to be a major mechanism which prompts tumorigenesis, and in fact, inflammatory cytokines and related cells are essential components of the tumor microenvironment. 21 Simultaneously, MMP-9 is known to cleave and activate tumor growth factors and trigger signal transduction pathways which inhibit cell apoptosis and induce increased proliferation rates. 22 A study by Schönbeck et al demonstrated that MMP-9 can cleave and, in turn, activate interleukin 1β (IL-1β) which is an inflammatory cytokine involved in many processes of inflammation. 23 Moreover, it has also been shown that MMP-9 can activate transforming growth factor-β (TGF-β) through proteolysis, and it is known that TGF-β is an inflammatory molecule that specifically acts as a bridge between inflammation and cancer. 24 25

On the contrary, MMP-9 is known to activate the vascular endothelial growth factor (VEGF) protein family and thereby promote angiogenesis in tumors. 26 In this manner, it assists the survival of malignant cells. Overall, MMP-9 can act as a procarcinogenic and pro-survival enzyme.

### Matrix Metalloproteinase-9 and Metastasis

**Fig. 1:**
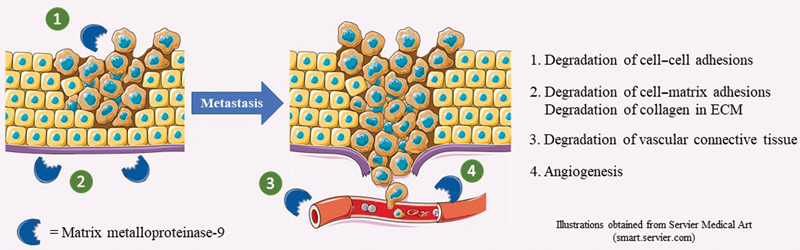
Potential prometastatic effects of MMP-9.

In the process of metastasis, one of the major barriers to malignant cell migration is the ECM, involved both in the process of tissue invasion and intravasation into the blood or lymph. 27 In this manner, MMP-9 due to its degrative nature in the ECM can assist tumors overcome this barrier. Specifically, the ability of MMP-9 to proteolyze type IV collagen in the basal lamina of tumors can alter the structure of the ECM and ease the invasion process. 28 29 Furthermore, MMP-9 can degrade the adhesive proteins on the surface of malignant cells responsible for cell-to-cell adhesions as well as the proteins which adhere cells to the ECM. 18 30 31 Thus, cells can be released more easily from the tumor tissue and metastasize. It is also worth mentioning that the connective tissues of blood and lymph vessels are rich in proteins, including type IV collagen and elastin, that can be degraded by MMP-9, and in this manner, the enzyme can assist the intravasation of cancerous cells. 32 On the contrary, as MMP-9 is an enzyme which can assist angiogenesis and activate VEGF in cancerous tissues, it can help support metastasis as more blood vessels are created adjacent to the tumor, and hence, malignant cells can have easier access to the bloodstream. 26 All potential mechanisms of action of the MMP-9 enzyme in tumor migration have been synopsized graphically in Fig. 1 .

## Polymorphisms of the *Matrix Metalloproteinase-9* Gene in Cancer

With cancer being a multifactorial disease, polymorphisms in different genes have been reported to highly increase the risk of tumorigenesis and poor prognosis. 33 Indeed, genetic polymorphisms in the *MMP-9* gene have been found to be correlated with a higher risk of carcinogenesis and susceptibility to neoplastic diseases. In Table 1 , the polymorphisms of the MMP-9 that have been shown to be correlated to oncogenesis in different types of cancer have been summarized.

**Table 1:** Polymorphisms of the *MMP-9* gene correlated with higher tumorigenesis and metastasis risk

Type of cancer	Polymorphism	Genetic model	Genotypes correlated with cancer	Polymorphism associated with tumorigenesis risk	Polymorphism associated with metastasis risk	References
Breast cancer	MMP-9-1562 C/T	Recessive	TT	✓	✓	34 35
Bladder cancer	MMP-9-1562 C/T	Recessive	TT	Unknown	✓	36
Colorectal cancer	MMP-9-1562 C/T	Recessive	TT	✓	✓	37 38
Gastric cancer	MMP-9-1562 C/T	Dominant	TT + CT	✓	✓	39
Lung cancer	MMP-9-1562 C/T	Dominant	CC + CT	✓	✓	40
Lymphoblastic leukemia	MMP-9-1562 C/T	Recessive	TT	✓	✓	41
Melanoma	MMP-9 2003 G/A	Recessive	GG	✓	✓	42
Oral cancer	MMP-9-1562 C/T	Dominant	CC + CT	✓	Unknown	43 44
Ovarian cancer	rs6094237 (T/A)	Dominant	AT + AA	✓	Unknown	45
Prostate cancer	MMP-9-836 A/G	Recessive	AA	✓	Unknown	46
Renal cancer	R279Q A/G	Dominant	GG + AG	Unknown	✓	47
Thyroid cancer	MMP-9-1562 C/T	Dominant	CT + TT	✓	✓	48 49

The well-known MMP-9-1562 C/T promoter single-nucleotide variation (SNV) is found to be associated with a higher risk of developing many types of cancer with the T-allele being the risk factor, except in the case of lung carcinoma where the wildtype C-allele is found to be correlated with a higher risk of cancer. 40 The MMP-9-2003 G/A and MMP-9-836 A/G SNVs have been shown to be correlated with a higher risk of melanoma and prostate cancer development respectively. 42 46 Another new polymorphism related to tumorigenesis, which has only been discovered in the case of ovarian cancer is the rs6094237 SNV that has also been shown to affect the vitamin-C receptors. 45 50

On the contrary, due to the prometastatic effects of MMP-9, polymorphisms in the respective gene may also lead to poor prognosis in many types of cancer. 51 Interestingly, the MMP-9-1562 C/T and MMP-9 2003 G/A polymorphisms are also associated with a higher risk of cancer migration in various types of cancer. 36 42 Another polymorphism known as R279Q A/G, which is located in exon 6 of the *MMP-9* gene, is associated with a higher risk of aggressive renal cancer and has been shown to be correlated with the histologic grading of renal cancer. 47 It is also worth mentioning that the T-allele of the MMP-9-1562 C/T polymorphism has been found through studies of other diseases to be associated with higher levels of MMP-9 enzyme expression. 52 Therefore, this polymorphism would rationally assist a quicker degradation of the ECM, leading to a higher risk of metastasis. Overall, the evidence from different studies suggests that the polymorphisms of the *MMP-9* gene can be used as a potential diagnostic and prognostic genetic marker.

## Regulation of the *Matrix Metalloproteinase-9* Gene Expression in Cancer

Different studies have discovered that the levels MMP-9 enzyme is increased in tissue samples and body fluids of cancer patients. 18 This indicates that the expression of the *MMP-9* gene is upregulated in cancer patients, which rationally is due to its significant role in the tumor microenvironment. Generally, the expression of MMP-9 is regulated through a variety of genetic mechanisms such as transactivation, as well as epigenetic mechanisms including DNA methylation. 53

To begin with, different cytokines and chemokines that are expressed in inflammation and cancer can initiate the synthesis of MMP-9 in different tissues. Indeed, cytokines including IL-1β, IL-17α, and tumor necrosis factor-α have been shown to induce the upregulation of the *MMP-9* gene by triggering signal transduction pathways that lead to the activation of transcription factors in different types of cancerous cells. 54 55 56 It has been demonstrated that the MEK/ERK signaling pathway is the main pathway responsible for inducing MMP-9 expression by activating the p38 protein which in turn acts as a transactivator in DNA transcription. 53 55 57 Furthermore, the mutations in the *Ras* gene that is involved in the oncogenesis process of multiple types of cancer have been shown to increase the activity of the MEK/ERK signaling pathway. 58 59 In this way, the mutation can promote carcinogenesis and metastases indirectly through the *MMP-9* gene.

Simultaneously, studies have shown that the *MMP-9* gene is hypomethylated in the case of breast cancer, Ewing's sarcoma and lymphoma cells, and at the same time the gene is upregulated. 60 61 62 Indeed, the methylation of DNA inhibits the binding of transcription factors, and therefore, hypomethylation of the gene induces upregulation of its expression. 63 Also, as mentioned previously, the polymorphisms of the *MMP-9* gene have also been found to be correlated with higher expression levels of the gene, but the underlying cause of this correlation is still not known. 52

Overall, all the available evidence indicates that since MMP-9 has a significant role in tumorigenesis and metastasis, one of the possible ways of controlling cancer and metastasis would be to either to inhibit the enzyme directly or inhibit the pathways that lead to the upregulation of the gene. In fact, numerous recent research has been conducted to use MMP-9 as an essential target in medical oncology. 53 Nonetheless, targeting MMP-9 directly has been found to have adverse side effects. 64 Thus, targeting the regulation mechanisms such as the MEK/ERK signaling pathway as an alternative approach can be a promising method that is being considered by many researchers. 65 66 67

## Conclusions and Future Perspectives

As seen in this review, the *MMP-9* gene plays a very significant role in cancer generally and various research has been undertaken in recent years to study the gene in more depth. On the one hand, the expression levels of MMP-9 as well as different polymorphisms of the gene have been found to be associated with higher risks of tumorigenesis and poor prognosis in cancer patients. Therefore, the *MMP-9* gene can be used as a potential genetic marker that may be able to assist pathologists and medical oncologists in earlier diagnosis of cancer, which will result in better treatment response, as well as predicting the risk of cancer migration. It is worth mentioning that genetic markers of cancer are of paramount significance in the field of oncology in today's era of precision medicine. 68 Nevertheless, for supporting the use of MMP-9 and its polymorphisms as a genetic marker, more clinical trials need to be undertaken.

On the other hand, due to the broad spectrum of the enzyme's action in the development and the progression of neoplastic diseases, the *MMP-9* gene as well as its expression mechanisms can be used as potential targets for cancer-targeted therapy. The monoclonal antibody andecaliximab is being studied through different clinical trials as a potential inhibitor of MMP-9; nonetheless, further clinical trials need to be performed before validating its efficacy. Nonetheless, other targets including those which interfere with the expression of MMP-9 can also be used in anticancer therapy. 69 Overall, clinical trials which study the effects of MMP-9 direct or indirect inhibition on the prognosis of patients are required to validate MMP-9 as a target for cancer therapy. A deeper study into the regulation of the *MMP-9* gene may also assist the discovery of novel agents which can control tumor progression by inhibiting the expression of MMP-9.
